# Remaking “The Unconscious” Paradigm, Act I: ...The Gap” Between Subliminal Processing and the Unconscious “Mind...

**DOI:** 10.1007/s12124-026-10048-7

**Published:** 2026-11-04

**Authors:** Myron Tsikandilakis, Persefoni Bali, Roland Erich Uriko, Mohammad Eskandari, Victοria-Maria Pasachidou, Romina Leonor Toranzos, Konrad Szczesniak, Ivona Dömischová, Pierre-Alexis Mével, Christopher Madan, Alison Milbank

**Affiliations:** 1 School of Psychology, University of Nottingham, Nottingham, UK; 2 Medical School, Faculty of Medicine and Health Sciences, University of Nottingham, Nottingham, UK; 3 School of Cultures, Languages and Area Studies, University of Nottingham, Nottingham, UK; 4 Institute of Psychology, University of Tartu, Tartu, Estonia; 5 Department of Psychology, Faculty of Psychology and Education, University of Tehran, Tehran, Iran; 6 School of Science and Technology, Nottingham Trent University, Nottingham, UK; 7 Estudos da Linguagem, Universidade Estadual de Londrina, Londrina, Brazil; 8 Institute of Linguistics, University of Silesia, Katowice, Poland; 9 Institute for Foreign Languages, Palacký University, Olomouc, Czechia; 10 Department of Philosophy, University of Nottingham, Nottingham, UK

**Keywords:** Subliminal, Unconscious, Methods, Models, Concepts

## Abstract

The notion of subliminal processing often refers to perception that takes place below the threshold of conscious visual awareness. This is suggested to lead to behavioural and emotional changes with spontaneous and actionable evolutionary-survival and/or social-adaptation response value. Therefore, it has been suggested that the presentation of imperceptible visual elicitors can lead to responses, such as skin-conductance and heart-rate, and facial-emotional, and participant behavioural and self-report changes without conscious awareness. In a series of experiments, we disproved this theory. We showed that individually-adjusted, truly imperceptible elicitors did not induce any of these changes, when using null-hypothesis-testing, and, furthermore, provided evidence for null responses, when using Bayesian analyses. We proceeded to a series of novel and exploratory experiments on unconscious theory. We tested whether behavioural and emotional responses were reported to precede conscious awareness using overt presentations of visual-emotional elicitors. We showed evidence that the experience of physiological-emotional responses was reported to precede conscious awareness of a perceived visual-emotional content. These early responses were automatic and involuntary; they did not involve or require conscious access, or accessibility, and deliberation, and could not be physiologically restrained by conscious instruction for inhibition. We proposed that responses that can be termed unconscious, but not subliminal, are preceding, spontaneous and conditionally uninhibited experiential components of exposure to perceived, but not imperceptible, emotional elicitors. We extended that they involve genuine evolutionary-survival and social-adaptation value, compared to so-called subliminal processes, because they enable spontaneously-occurring, and progressively-intentional and intended behavioural and emotional adjustments, and readjustments, and adapting refinements.

## Introduction

### The Genealogy of a Vastly Prevalent Model-Method

The unconscious, as a concept signifying a state, function, or behaviour in the absence of conscious awareness, has a very long and varying history. It begun, in written language, with ancient Greek philosophy, such as Heraclitus’ concept of *the waking slumber*, that suggests that certain people are oblivious of their own selves, and alike sleeping while awake (521–511 BCE), and Plato’s concept of innate knowledge, as *anamnesis* (385–380 BCE; see Crivellato & Ribatti, 2007; Tsikandilakis et al., 2026). It continued to transcendental philosophy, such as Schelling’s notion of the sense of an inner archaic and primordial, immemorial unity with the world, and Hartmann’s notion of a primordial and pre-ontological force that precedes and results in materialisation (Otabe, 2019). It later extended to Freud’s strictly sexually-driven psychoanalytic model of repression, Aaron Beck’s unawareness of the *vicious circle* of recurring harmful behaviours in clinical psychopathology (Mills, 2014), and even Vicary’s proven hoax that he increased certain product sales by presenting very brief suggestive cues to promote beverage and pop-corn products, in random intervals during movies in cinema theatres (Tsikandilakis et al., 2025a). From the aggregate plurality of all these conceptualisations and applications, we have come to an era when the unconscious is near monopolised as “The Subliminal” in contemporary psychological research, and especially in its testing via visual suppression (Tsikandilakis et al., 2019a). This prevalent model-method poses two significant problems. First, as scholars and scientists from Heraclitus to Darwin, and even Freud, anterogradely, in this instance (Donald, 2001), and contemporary researchers (Seth & Baars, 2005) have suggested, subliminality involves a paradoxical claim: Imperceptible emotional elicitors lead to imperceptible emotional elicitation. This is a philosophical and evolutionary paradox: It returns the workings of the unconscious to Heraclitus’ notion of *a waking slumber*, and would be a hurdle to human evolution; it would disallow consciousness to emerge in any form, and cognition and actionable behaviours to be adapted, adjusted, and refined, and would render even the planning and achievement of historically-recorded and contemporary-accomplished technological advancements unattainable (see Eccles, 1992). Secondly, irreverently to the consideration of any conceptual or scientific canon, contemporary applications of subliminal presentations are performed as James Vicary performed his infamous hoax. Vicary used the lowest frame-rate of a high-end cinema projector of his time, presenting purportedly imperceptible stimuli for 20 ms (monitor set at 50 Hz). Researchers today commonly use the lowest frame-rate of a standard laboratory monitor to present purportedly imperceptible stimuli for 17 ms (monitor set at 60 Hz). We have previously discussed these concerns, and other problems of “The Subliminal Paradigm”, and included thorough references to people who have done the same (Kouider & Dupoux, 2004), and yet the paradigm stands; reviews accept the validity of our claims, as much as applied research continues to ignore them (Tsikandilakis et al., 2021a, 2023a; Uriko et al., 2026). Now, it is the time for a different and more provocative kind of illustration, but first, let us provide – beyond our own objections – an account, as objective as possible, of the unconscious in contemporary psychological research, starting with “The Subliminal” and subliminal processing as an applied and conceptual paradigm.

#### “The Subliminal”, A Vestly Prevalent Model-Method

Subliminal processing can be defined as participant responses to imperceptible emotional stimuli. Emotional stimuli can be rendered imperceptible using visual suppression techniques (Bruce et al., 2003; Dubois & Faivre, 2014). One widely used visual suppression technique is backward masking. Backward masking can include a very brief presentation of a target-elicitor (e.g., ≥ 7 to ≥ 50 ms) followed by a noise mask (i.e., ≥ 100 ms). After the presentation, participant responses are recorded to explore whether imperceptible stimuli induced physiological changes. The methods that are used for this purpose can include peripheral nervous system (PNS) assessments, such as subcutaneous sweating responses (SCR), heart-rate responses (HR), and facial-emotional expression analyses. These are suggested to result in changes when participants are exposed to different types of imperceptible emotion. Central nervous system assessments (CNS), such as functional magnetic resonance imaging (fMRI), electroencephalography (EEG) and functional near-infrared spectroscopy (fNIRS) can also be employed. These can provide results for whether emotion processing brain structures were activated as a result of the presentation of imperceptible emotional elicitors (see Cacioppo et al., 2007; pp. 587–99).

The mechanism that has been suggested to result in responsivity to imperceptible stimuli is subliminal perception. Subliminal perception is based on the *dissociation paradigm,* such as making an elicitor imperceptible, and recording imperceptible emotional changes in response to this imperceptible elicitor (Boag, 2008). In psychophysical terms, this can be interpreted to signify that when perception of a stimulus is defined as α, and responsivity to a stimulus is defined as ε, then subliminal perception occurs when ε > α | α = 0 (Erdelyi, 2004). The definition of α = 0 should be performed using objective measures for proving null perception, such as Bayesian analyses for equivalence to the null (Dienes, 2016), and unbiased metrics for imperceptibility, such as signal-detection-theory (SDT) sensitivity index A (Zhang & Mueller, 2005; see also *Experiment One: Statistical Framework*). Additionally, evidence for ε > α should be exclusive to instances of α = 0 (Overgaard & Timmermans, 2009), such as incorrect responses – also referred to as false negative responses (FN) – for not perceiving a stimulus in a post-trial, or post-experimental signal-detection task (see Hautus et al., 2021).

From an evolutionary psychology perspective, subliminal perception is suggested to occur via archaically retained cognitive mechanisms that confer phylogenetic survival-related and sociobiological value (Liddell et al., 2005; Öhman et al., 2007). This is proposed to occur through a dedicated subcortical neural pathway with PNS arousal dissemination functions. Relevant research has provided evidence that a direct pathway from the visual thalamus to the amygdala bypasses the occipital (visual) cortex and innervates a cascade of temporal lobe structures. These structures can include the hippocampus and the fusiform gyrus, and eventually reach the brainstem and the cerebellum. These are suggested to induce top-down PNS responses, such as increased SCR and HR responses, and elicit bodily sensations associated with emotion. Evidence for these effects have been cited by meta-analyses using fMRI, EEG and FNIRS (see Brooks et al., 2012; pp. 2965 & 2967; Dahlen et al., 2022; pp. 3–4; Meneguzzo et al., 2014; pp. 4 & 7), and supported by meta-analyses citing PNS assessments, such as SCR and HR responses (see van der Ploeg et al., 2017; pp. 139–141).

#### “The Unconscious Mind”, A Differential Concept-Model

Another interpretation of what unconscious responses represent, and how they operate, is the notion of “The Unconscious Mind”. This concept involves the ideas that automatic and involuntary CNS and PNS responses contribute to ecologically necessary cognitions and actions (for a relevant review, see Pessoa & Adolphs, 2010). These responses are suggested to be physiologically uninhibited but behaviourally inhibitable. They are suggested to take temporal primacy to, but to interact with, and to eventually recruit conscious awareness. This is due to that part of the purpose of these responses is conscious accessibility, such as stimulus sensitivity, and access, such as stimulus awareness, that can facilitate real-life actionable cognition and priming, and skill-acquisition (Bargh & Morsella, 2008). The unconscious mind model is based on the *new look social paradigm* for the psychological unconscious, such as that real-life ecological survival and social adaptation can be achieved through the temporal primacy of unconscious emotional responses that eventually interact and inform consciousness awareness to facilitate a best-appropriate behavioural response-strategy. Therefore, this model suggests that unconscious responses take temporal primacy to perceptible emotional elicitors, and does not support the ecological validity of masked elicitors, or dissociated emotional responses. On the contrary, it supports that masked presentations and dissociated responses have no ecological validity because they are not encountered in real-life environments, and that unconscious responses take temporal primacy when participants are exposed to perceptible emotional stimuli, and are further manifested and processed by conscious behavioural guidance functions (see Bargh, 2013; see also *Experiment Two: Objectives & Statistical Outcomes*). In psychophysical terms, “The Unconscious Mind” model can be interpreted to signify that for τ(χ) and τ(y) being two distinct points in time, unconscious responses occur when ε_τ(x)_ ≺ α_τ(y)_ | α > 0. This suggests that the unconscious mind represents a system of emotionally automatic and involuntary, and physiologically-driven responses to perceivable, and eventually consciously accessible, and behaviourally adjustable, but not imperceptible and dissociated, emotional experiences (see Foglia & Wilson, 2013).

From an evolutionary psychology perspective, the existence of an unconscious mind confers actionable priming survival value. This suggests that the workings of the unconscious mind can involve, recruit, and retain for further employment, higher executive processes and conscious functions, such as adaptable memory-encoding-and-retrieval priming mechanisms (Tsikandilakis et al., 2018, 2022). This can result in unconscious emotional responses, very fast cognitions and behaviours, and behavioural cultivation and interactions between phenomenal, such as emotional, and cognitive, such as appraising, conscious functions, that can lead to progressively intentional and intended behavioural and emotional adjustments, and readjustments, and adapting-evolutionary refinements (see Bargh & Chartrand, 2000). This model is supported by findings that show that a dual two-way interactive stream of neuronal activation occurs in response to perceptible emotional elicitors. This parallel CNS cascade starts in the visual thalamus. Then it continues to the occipital cortex, and then it is suggested to split into two pathways. A faster route is suggested to connect via the occipital cortex to key limbic lobe areas, such as the amygdala. Subsequently, it is suggested to innervate the cerebellum and the brainstem and elicit automatic PNS responses. This pathway is suggested to induce automatic and involuntary bodily sensations associated with emotion. A slower pathway is suggested to follow a dorsal route from the occipital cortex via mid-parietal cortex areas to the frontal and, most critically, the prefrontal cortex (for a relevant review, see Madan, 2023; but see also Szcześniak, 2024; Szcześniak et al., 2026). This pathway is suggested to confer meta-awareness, such as eventual conscious phenomenal self-awareness of the bodily sensations associated with emotion, and meta-cognition, such as eventual conscious appraisal of the elicitor, or elicitors, related to an emotional experience (Jost et al., 2013). This model is supported by evidence cited in meta-analyses using fMRI, EEG and FNIRS, and PNS assessments (see Gronchi et al., 2024; pp. 5–7 & 14; Jobard et al., 2003; pp. 695 & 699–703; Nieuwenstein et al., 2015; pp. 8–11; see also Evans & Stanovich, 2013; pp. 225–9).

### Testing the Paradigm(s)

The workings of subliminal processing and perception, and the unconscious mind are currently, and have been for at-least the past fifteen years, an issue of contention for psychological science (see Berlin, 2011). These two models have been conceptualised as conflicting accounts for applying to and explaining our unconscious processes and responses (Tsikandilakis & Chapman, 2018; Tsikandilakis et al., 2019b, 2020a). They are considered to involve mutually exclusive properties and characteristics. These remain unresolved. They include the requirement of subliminal perception, typically via masking, to procure unconscious responses, versus the rejection of research including masked emotional elicitors, due to the ecological impossibility of encountering real-life masked emotional stimuli. They include, critically, the proposition that imperceptible elicitors lead to imperceptible cognitive, emotional, and behavioural outcomes, versus the proposition that perceptible emotional elicitors lead initially to unconscious emotional responsivity, and subsequently, via self-perception, to conscious phenomenal-emotional. cognitive, and behavioural awareness and regulation (see Borsboom et al., 2021; pp. 3–5).

The explorative objective of the series of studies in the current manuscript was to test and contrast the validity of the resulting outcomes for these two models (see Kline, 2005; pp. 91–94). To this end, we conducted a total of three experiments. In Experiment One, we applied the method of individual subliminality (Engelen & Mennella, 2023; Fisk & Haase, 2025; Jimenez et al., 2023; Schreiber et al., 2025), including contemporary state-of-the-art innovations for this method by the corresponding lab (Tsikandilakis et al., 2020b), to explore whether truly imperceptible emotional faces can induce changes in PNS and behavioural responses, and self-reports (Jimenez et al., 2025). In Experiment Two, we tested whether overt emotional faces were effective elicitors for PNS physiological, and behavioural correlates of emotion, and we applied timescale analyses to explore whether physiological experience and conscious perception showed temporal primacy differences during the processing of these presented faces (Wilson & Golonka, 2013). In Experiment Three, we proceeded to the novel and potentially extensive to the paradigm forming of “The Unconscious Mind” exploration of whether PNS and behavioural responses could be primed and inhibited (see Mulckhuyse & Theeuwes, 2010; Prasad & Mishra, 2019; Rohr & Wentura, 2021). We tested the physiological and behavioural correlates of overt emotional faces under four pre-trial labelling conditions: No pre-trial label, pre-trial mention that a stimulus would be presented, explicit instruction for attentional focus, and explicit instruction for physiological inhibition (Tsikandilakis et al., 2020c, 2024; 2025b).

### The Hypotheses

For Experiment One, as per our introductory rationale (see Section "Introduction"), and previous research (Tsikandilakis et al., 2023a), we anticipated that after individually adjusting subliminal presentation per participant and stimulus type to chance-level performance, using objective and unbiased non-parametric sensitivity index A, and Bayesian analyses for equivalence to the null, we would be able to achieve a true criterion of α = 0, and we would not be able to report null-hypothesis-significance-testing (NHST) evidence, and we would be able to report Bayesian evidence for the null, for physiological and self-reported differences among the presented emotional elicitors, thus disproving the “Subliminality” paradigm. For defining chance-level performance individually using potentially biased criteria for α = 0, such as hit rates that can be subject to liberal and conservative response strategies (see Stanislaw & Todorov, 1999; pp. 137–139), we anticipated among-stimuli differences. Nevertheless, we anticipated that exhaustive and exclusive analyses of SDT responses would not provide evidence for subliminal processing. SDT responses are separated among false negatives (FN), such as incorrectly responding that a stimulus was not presented, true negatives (TN), such as correctly responding that a stimulus was not presented, false positives (FP), such as incorrectly responding that a stimulus was presented, and true positives (TP), such as correctly responding that a stimulus was presented (see Macmillan, 2002; pp. 46–49). We anticipated that for biased hit-rate individual adjustments for purportedly subliminal presentations, only TP and FP would provide evidence for differences among stimulus types, indicating that either conscious perception, or misperception, comprised the reported effects (Tsikandilakis et al., 2025b).

For Experiments Two and Three, we anticipated to show that perceptible emotional faces – as proposed to be presented during the experimental trials by “The Unconscious Mind” model-method paradigm (see Bargh & Morsella, 2008; pp. 2–5) – would be effective physiological and behavioural elicitors. Having established that, for Experiment Two, we anticipated that, if the conceptual and applied corelates of “The Unconscious Mind” paradigm are correct, such as that a dual neural cascade including a ventral pathway for the dissemination of arousal precedes CNE structures related to conscious awareness, participants would report physiological experience as preceding the conscious perception of emotional faces when using timescale analyses. Conversely, in Experiment Three, we anticipated that physiological changes in response to emotional faces could not be regulated by conscious instruction for emotional inhibition, and that behavioural responses, such as facial-emotional expressions, could. We expected emotional outcomes for evolutionary-adaptive priming responsivity, including behavioural responses, such facial-emotional expressions, to show an opposite pattern of outcomes as an end result of interactive adjustments between initial unconscious responses and eventual conscious accessibility, and re-enhancement (see Baars et al., 2003).

## Experiment One: Individual Subliminality

*Objectives:* The objectives of the current experiments were to assess SCR, HR, facial-expressive and self-report responses to biased and unbiased individually-adjusted for subliminal presentations emotional faces.

*Participants:* A power calculation based on a univariate within-subjects design and small effect sizes (f =.1; d =.2) given the current experimental trial contour (K _stimulus type_ = 60/20; see *Experimental Procedures*) revealed that 282 participants were required for P _(1- β)_ ≥.9 (*p* ≤.01, p (H1) ≥.9; B > 3; f [≥.1, + ∞]; Tsikandilakis et al., 2019c, 2021b, 2023b).

In a preliminary session, one week before the first session of the main study, 329 volunteers were screened without prior knowledge of the experimental objectives. The exclusion criteria were that participants should not have or have been previously diagnosed with a DSM Axis I or II disorder (American Psychiatric Association, 2022), and current or previous alcohol/drug abuse; self-report (Y/N). For further validation, the participants were screened with questionnaire assessments: Somatic and Psychological Health Report Questionnaire (SPHRQ; Berryman et al., 2012), Stressful Life Events Screening Questionnaire (SLESQ; Allen et al., 2015), Moral Injury Questionnaire (MIQ; Koenig & Al Zaben, 2021), Personality Disorder Questionnaire (PDQ; American Psychiatric Association, 2022), Emotional Reactivity Scale (ERS; Nock et al., 2008) and an online multiple-trait component Alexithymia/Emotional Blindness questionnaire (AQ; Alexithymia, 2024). The preliminary session lasted for 25–30 min. After the completion of the screening, the participants were asked whether they would be interested to participate in a further two-stage study relating to facial-emotional processing (see Tsikandilakis et al., 2025c).

Forty-one participants who fitted the criteria for inclusion did not volunteer to further participate in the subsequent study. Data from three participants were excluded from further analysis due to SPHRQ scores that indicated a possible psychiatric diagnosis. Data from two participants were excluded from further analysis due to PDQ scores that indicated a possible personality disorder. Data from a single participant were excluded from further analysis due to AQ scores that indicated Alexithymia traits. The population sample consisted of 282 participants (141 females) with mean age 27.91 (SD = 4.27) years. The mean age for males and females was 27.16 (SD = 4.98) and 28.66 (SD = 3.56) respectively. The experiment was approved by the Ethics Committee of the School of Psychology of the University of Nottingham.

*Stimuli*: 500 facial expressions (n _actors_ = 100; k _expression per actor_ = 5) were used from the dataset created by Gur and colleagues (2002). The stimuli were selected based on that they showed non-significant differences and provided Bayesian evidence for equivalence of significance to the null for within-stimulus-type-category SCR and HR responses, and emotional categorisation, and additional facial characteristics, such as intensity, valence and salience, and attractiveness, and racial and cultural familiarity ratings in several previous publications (Tsikandilakis et al., 2018, 2019b; 2019c; 2021b; 2023b). Non-facial masked blurs were generated using pseudo-randomised pixel permutation in MATLAB. Black-and-white pattern masks were created in MATLAB using random chess-board-box permutation. The facial stimuli were adjusted for interpupillary distance. All stimuli were transformed to grey scale and resized to 1024 × 768 pixels. Their luminance was averaged using the SHINE, MATLAB Toolbox, and they were spatially aligned and placed in a cropped circle with set dimensions (height: 6 cm, width: 4 cm; Mask tile-size periphery =.048 cm).

*Experimental Procedures:* Before the beginning of the main study, a five-minute on-screen tutorial was presented to the participants that showed them how to operate the keyboard and mouse components included in the experiment, and presented with explanations, and definitions relating to the terminology used in the experiment. Participants were asked whether they understood the instructions and were encouraged to ask the experimenter concerning any queries. This tutorial stage was repeated for every experimental session in this paper; no instances of negative responses, or participant queries were reported (see Experiments Two and Three).

The main study consisted of two experimental sessions. In the first experimental session, each trial started with an on-screen fixation cross for two (± one) seconds. After the fixation cross, a single face showing fear or anger or happiness or sadness, or a neutral expression, or a blur was presented at fixation for 6.94 or 13.89 or 20.83 ms (i.e., monitor set at 144 Hz) with duration of presentation order randomized (k _facial type_ = 60; k _facial type/gender_ = 30; k _facial type/gender/duration_ = 20; k _blurs_ = 300). The image was immediately followed by a black-and-white pattern mask for 104.17 ms. Then a blank screen was presented for five seconds.

After the blank-screen interval, the participants were asked whether they saw a face during the presentation (“A” for Yes, “S” for No; key order randomised). Subsequently they were asked to rate, from one (not at all) to nine (very) how confident they were with their reply. After the questions, a blank-screen interval was presented for five seconds, before the next trial. The signal-detection question was used to define per participant and stimulus type the duration (i.e., 6.94 or 13.89 or 20.83 ms) that provided Bayesian evidence (BF <.33) for equivalence to the null (chance-level performance) at A =.5 [CIs: LB =.4 and UB =.6] and hit rates (HRs) = 50% [CIs: LB = 40% and UB = 60%] (for the methodology of definition of a-priori CIs, see Dienes, 2021a, 2021b; see also *Statistical Framework*).

Session two took place a week after at the same timeslot as session one. It consisted of two parts (order randomised) separated by a five-minute break. In one part, participants were presented with masked stimuli individually adjusted per participant and stimulus type to the duration that during session one provided Bayesian equivalence to chance-level perceptual performance when using unbiased sensitivity index A. In one part, participants were presented with masked stimuli individually adjusted per participant and stimulus type to the duration that provided equivalence to chance-level perceptual performance when using HRs. For each part of the second session, each trial started with an on-screen fixation cross for two (± one) seconds. After the fixation cross, a single face or a blur was presented at fixation for 6.94 or 13.89 or 20.83 ms (k _facial type (part 1 or 2)_ = 100; k _facial type (part 1 or 2)/gender_ = 50; k _blurs (part 1 or 2)_ = 100). Each face or blur was immediately followed by a black-and-white pattern mask for 104.17 ms. A blank screen was presented for five seconds during which facial-expressive and physiological responses were recorded.

**Fig. 1:**
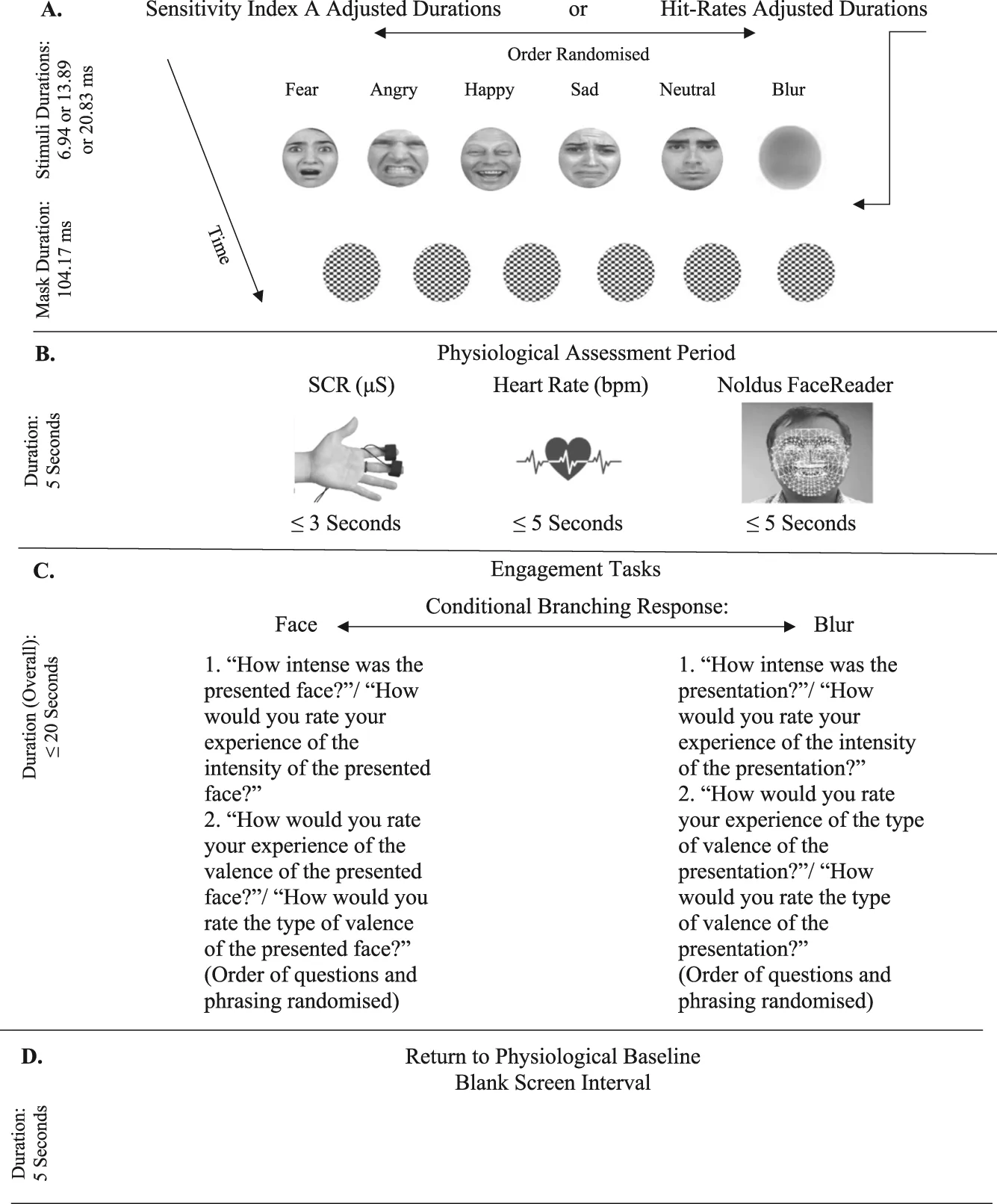
Experimental design, experiment one, session two. In **A**., adjusted backward masked faces presented for 6.94 or 13.89 or 20.83 ms (K _stimulus type_ = 20), defined during experimental session one for the duration that for each participant and for each stimulus type provided Bayesian evidence (B <.33) for statistical proximity to chance when using sensitivity index A (.5) and hit-rate (50%) metrics. In **B**., SCR, HR and Noldus Face Reader assessments for a period of three to five seconds post-stimuli offset was allowed to assess psychophysiological responses. In **C**., an illustration of the period during which the participants were asked whether they saw a face or a blur, and were engaged in self-report tasks. In **D**., a period of five seconds was allowed for physiological responses to return to baseline

After the blank-screen interval, participants were asked to respond to a set of questions using the keyboard or mouse. They were asked whether they saw a face during the presentation (“A” for Yes, “S” for No; order randomised). Subsequently, they were asked to rate, from one to nine, how confident they were with their reply. Using conditional branching, further questions were adapted accordingly. If a participant replied “Yes”, they were asked to rate from one (not at all/very negative) to nine (very/very positive) “How intense was the presented face?” and “How would you rate your experience of the type of valence of the presented face?”; order of questions and phrasing randomised. Using further conditional branching, if they replied “No”, the participants were asked, “How intense was the presentation?”, and “How would you rate your experience of the type of valence of the presentation?”; order of questions and phrasing randomised. After the questions, a blank-screen interval was presented for five seconds (see Tsikandilakis et al., 2025a, 2025b, 2026; see also Fig. 1).

**Fig. 2:**
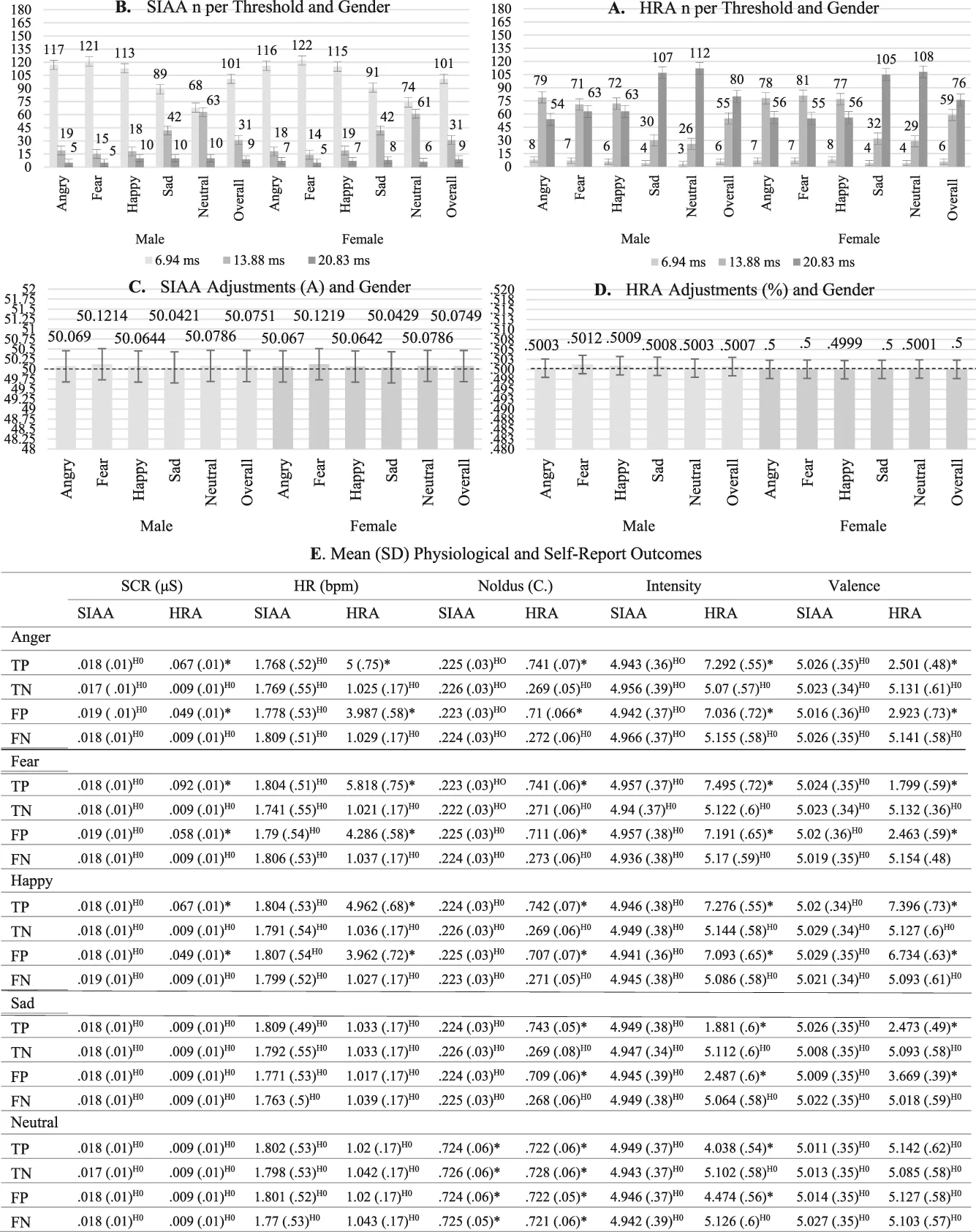
Experimental outcomes, experiment one. In **A**., **B**., **C**. and **D**. signal-detection performance for SIAA and HRA adjustments; data are presented with stimulus-type specific error bars indicating ± 2 SDs of each measure of each mean, or count. In **E**., participants’ responses per signal detection item (TP, TN, FP and FN) for SIAA and HRA adjustments.* (Asterisk) shows Bonferroni-corrected significance at *p* ≤.001, and Bayesian evidence for the alternate at B > 3. ^H0^ (superscript) signifies evidence for equivalence of significance to the null (see Dienes, 2021a; 2021b)

*Statistical Framework*: The statistical analyses were performed using both NHST and Bayesian inference. NHST was set at *p* ≤.05 for omnibus ANOVA tests with post-hoc comparisons with Bonferroni corrections ($\frac{\upalpha }{\mathrm{k}}$). Greenhouse–Geisser corrections were applied throughout the analyses for violations of sphericity for repeated-measures ANOVAs (see Lakens et al., 2018). Bayesian analyses were based on an a-priori minimum effect sizes of interest paradigm for lower and higher-bound, such as 40% and 60% for lower and higher-bound for hit rates, and.4 and.6 for sensitivity index A (for thorough reviews, and experimental precedence, on this subject, see Dienes, 2016, 2021a, 2021b; Tsikandilakis et al., 2019a, 2021a). There was no research precedence for individual subliminality, as applied here (see Tsikandilakis et al., 2023b, 2025a, 2026), and timescale analyses applications for conscious versus unconscious processing models (see Jimenez et al., 2025; see also Fisk & Haase, 2025; Fisk, 2026; Haase, 2026). Accordingly, for Bayesian analyses, evidence for the null hypothesis were defined at a Bayes Factor (BF) <.33 using a uniform distribution with priors for a minimum effect size of interest model of BF (H0) at f [0, ≤.05]. Evidence for the alternate hypothesis was defined at BF > 3 using a uniform distribution with priors adjusted for a minimum effect size of interest model of BF (H1) at f [≥.1, + ∞). Intermediate results were considered inconclusive at.33 < BF(H0-1) < 3 (see Eberly & Casella, 2003; pp. 121–127; see also Armstrong et al., 2022; Angers & Biswas, 2003; Dienes, 2021a, 2021b; for statistical parameters, see Fig. 2, Tables 2 and 3 & Appendix 1–3). All Bayes factors were calculated and can be replicated using the online calculator https://harry-tattan-birch.shinyapps.io/bayes-factor-calculator/ (see Chen et al., 2023). Sensitivity index A was used as an objective index of participants’ perceptual performance. It was used because A, compared to d’, is a non-parametric index, and does not involve any requirements or assumptions for the shape of the underlying signal-to-noise likelihood distributions. Furthermore, sensitivity index A involves diagonal Euclidean corrections for scores that lie in the upper left quadrant of the ROC space (i.e., False Alarms Rate ≤.5 & Hit Rate ≥.5) that are not included in non-parametric indices A’ and A'’ (see Zhang & Mueller, 2005; pp. 203–7). This statistical framework was applied throughout the experiments in this paper (see Tsikandilakis et al., 2021a, 2023b, 2025a).

*Facial-Expressive Responses Framework:* Analysis of the participants’ facial expressions was conducted using Noldus FaceReader 9.1. An UHD (4 K) CANON camera positioned at the bottom of the screen was centred on the participant’s face. The analysis was conducted using the highest video capture frames per second allowed by the equipment (thirty fps) and employed a 500-points digitally imposed Euclidean network upon the participants’ faces (see Zaharieva et al., 2024; pp. 5712–3). The full property modules of Noldus FaceReader were used to eliminate static identification variability for image quality, lighting, background variation, and face orientation. Each participant was evaluated as regards to an expressed emotion after controlling for action units that were present in their own neutral expressions using the individual-participant calibration module (see Zaharieva et al., 2024; pp. 5710–1). Facial-emotional recognition was defined strictly as a categorical classification criterion (C _discrimination_ ≥.7) for the expression of emotion (see Küntzler et al., 2021; pp. 4–7). The participants were aware that their facial expressions were recorded. This framework was applied throughout the experiments in this paper (see Tsikandilakis et al., 2024; 2025a; 2025b).

*Physiological Assessment Framework:* SCR and HR were assessed simultaneously. SCR was measured from the left hand (index/first and middle/second fingers) of each participant using disposable Ag/AgCl gelled electrodes. The signals were received by a BIOPAC, EDA100C in units of microsiemens (μS) and recorded in AcqKnowledge. HR was measured via a single-finger sensor from the left hand (ring/third finger). The signal was received by a BIOPAC, PPG100C, using infrared photoplethysmography of blood flow fluctuations. The raw signal was recorded and converted in beats per minute (bpm) in AcqKnowledge. The presence of a phasic SCR was defined as an unambiguous increase (μS ≥.01) occurring up to three seconds post-stimulus offset (see particularly Cacioppo et al., 2007, pp. 163–4). The presence of an HR response was defined as an event-related heart-rate peak in beats per minute occurring up to five seconds post-stimulus offset (see particularly Cacioppo et al., 2007; pp. 187–8). The responses were calculated using the inbuilt derive phasic from tonic and find cycles AcqKnowledge routines as the highest peak in physiological responses (δ) in respect to a tonic baseline averaged across a period (δT) of each pre-stimulus onset using parallel port-input derived onset markers (see particularly Braithwaite et al., 2013; pp. 1019–21). This framework was applied throughout the experiments in this paper (see Tsikandilakis et al., 2020a, 2020b, 2020c).

*Results. Signal Detection*: Overall, signal detection performance was not significantly different.

to chance and provided Bayesian evidence for equivalence to the null for hit-rate (t (281) =.369, *p* =.779; d _standardised_ =.051; BF =.11) and sensitivity-index-A individual adjustments (t (281) =.496, *p* =.62; d _std_ =.067; BF =.21). This effect was consistent for male (t (140) =.779; *p* =.921; d _std_ =.06; BF =.09) and female participants (t (140) =.978, *p* =.982; d _std_ =.01; BF =.03), for every included stimulus type (t (846) = 1.094, *p* =.84; d _std_ =.046; BF =.09). These results showed that the application of individual thresholds was invariably successful during the first session of the experiment (Fig. 2A-D).

*Results. Psychophysiology*: For sensitivity-index-A-adjusted stimuli (SIAA), for SCR, no significant differences and Bayesian evidence for equivalence to the null were shown among Stimulus Types (Anger, Fear, Happy, Sad, Neutral, and Blurs) (F (5, 1400) =.298, *p* =.914, η^2^_p_ =.001; BF =.03), and no significant interactions were reported for Signal Detection Responses (TP, TN, FP, FN) (F (15, 4200) = 1.072, *p* =.378, η^2^_p_ =.004; BF =.08) and Gender (Male and Female) (F (5, 1400) =.342, *p* =.888, η^2^_p_ =.001; BF =.03). Similar results were revealed for heart-rate responses among Stimulus Types (F (5, 1400) =.642, *p* =.661, η^2^_p_ =.008; BF =.14), and for Signal Detection Responses (F (15, 4200) =.878, *p* =.59, η^2^_p_ =.003; BF =.06) and Gender interactions (F (5, 1400) =.903, *p* =.478, η^2^_p_ =.003; BF =.06). With respect to emotion recognition, participants only met the Noldus criterion (C discrimination ≥.7) for emotional expressions for neutral expressions in response to neutral faces (M =.87, SD =.01) without other significant differences among Stimulus Types (F (12.84, 3623.138) =.864, *p* =.788, η^2^_p_ =.002; BF =.03). These results showed that truly subliminal stimuli had no effect on the participants’ psychophysiological responses.

For hit-rate-adjusted stimuli (HRA), for SCR, significant differences were shown among Stimulus Types (F (1.188, 332.521) = 7121.434, *p* <.001, η^2^_p_ =.962; BF = + ∞) and significant evidence for Signal Detection Responses (F (5.062, 1417.347) = 21.502, *p* <.001, η^2^_p_ =.904; BF = + ∞) and Gender interactions (F (1.188, 332.521) = 12.57, *p* <.001, η^2^_p_ =.043; BF = + ∞). SCR scores were higher only for TP (*p* <.001; d _std_ = 6.89; BF = + ∞) and FP responses (*p* <.001; d _std_ = 4.46; BF = + ∞), while FN (*p* =.463; d _std_ =.017; BF =.17) and TN responses (*p* =.462; d _std_ =.018; BF =.17) did not show significant differences and showed Bayesian evidence for equivalence to the null. Overall, females (M =.037, SD =.001) showed higher SCR compared to males (M =.034, SD =.001; *p* <.001; d _std_ = 3.11; BF = + ∞). Similar results were revealed for heart-rate responses for Stimulus Types (F (3.618, 1013.136) = 12655.614, *p* <.001, η^2^_p_ =.978; BF = + ∞), and for Signal Detection Responses (F (7.437, 2082.82) = 4513.443, *p* <.001, η^2^_p_ =.942; BF = + ∞) and Gender interactions (F (3.618, 1013.136) = 144.757, *p* <.001, η^2^_p_ =.341; BF = + ∞). TP (*p* <.001; d _std_ = 2.828; BF = + ∞) and FP responses (*p* <.001; d _std_ = 2.623; BF = + ∞) showed significance, while TN (*p* =.723; d _std_ =.014; BF =.11) and FN (*p* =.741; d _std_ =.01; BF =.11) did not. Females (M = 2.029, SD =.143) showed higher scores compared to males (M = 1.849, SD =.156; *p* <.001; d _std_ = 1.219; BF = + ∞). Noldus FaceReader showed significant differences for Stimulus Types (F (4.783, 1339.357) = 9576.629, *p* <.001, η^2^_p_ =.972; BF = + ∞), and for Signal Detection Responses (F (5, 420) = 4.928, *p* <.001, η^2^_p_ =.18; BF = + ∞) and Gender interactions (F (4.783, 1339.357) = 26.436, *p* <.001, η^2^_p_ =.086; BF = + ∞). TP (*p* <.001; d _std_ = 3.628; BF = + ∞) and FP responses (*p* <.001; d _std_ = 3.191; BF = + ∞) showed significance, while TN (*p* =.719; d _std_ =.011; BF =.11) and FN (*p* =.721; d _std_ =.012; BF =.11) did not. Females (M =.791, SD =.023) showed higher scores compared to males (M =.711, SD =.026; *p* <.001; d _std_ = 3.527; BF = + ∞). These results showed that hit-rate adjusted stimuli induced physiological changes. These physiological changes were restricted to instances that participants perceived (TP), or erroneously believed that they perceived (FP), an emotional elicitor (Fig. 2E).

*Results. Self-Reports:* For SIAA, for intensity, no significant differences and Bayesian evidence for equivalence to the null were shown among Stimulus Types (F (5, 1400) =.649, *p* =.662, η^2^_p_ =.002; BF =.03), and no significant interactions were reported for Signal Detection Responses (F (15, 4200) =.967, *p* =.916, η^2^_p_ =.003; BF =.03) and Gender (F (5, 1400) =.798, *p* =.617, η^2^_p_ =.003; BF =.03). The same pattern of results was revealed for ratings for valence for Stimulus Types (F (5, 1400) =.713, *p* =.614, η^2^_p_ =.002; BF =.11), and for Signal Detection Responses (F (15, 4200) =.515, *p* =.934, η^2^_p_ =.002; BF =.07) and Gender interactions (F (5, 1400) =.408, p =.843, η^2^_p_ =.002; BF =.03). These results showed that subliminal stimuli had no effect in the participants’ rating responses.

For HRA, for intensity, significant differences were shown among Stimulus Types (F (4.7, 1316.008) = 7911.839, *p* <.001, η^2^_p_ =.966; BF = + ∞), and significant interactions for Signal Detection Responses (F (13.518, 3784.322) = 2825.252, *p* <.001, η^2^_p_ =.91; BF = + ∞) and Gender (F (4.7, 1316.008) = 42.416, *p* <.001, η^2^_p_ =.362; BF = + ∞). TP (*p* <.001; d _std_ = 2.911; BF = + ∞) and FP responses (*p* <.001; d _std_ = 2.872; BF = + ∞) showed significance, while TN (*p* =.232; d _std_ =.046; BF =.27) and FN (*p* =.241; d _std_ =.040; BF =.29) did not. Overall, females (M = 5.651, SD =.174) showed significantly higher scores for intensity compared to males (M = 5.019, SD =.168; *p* <.001; d _std_ = 3.073; BF = + ∞). For valence, significant differences were shown for Stimulus Types (F (4.743, 1327.962) = 4193.722, *p* <.001, η^2^_p_ =.937; BF = + ∞), and for Signal Detection Responses (F (13.318, 372.156) = 1578.637, *p* <.001, η^2^_p_ =.983; BF = + ∞) and Gender interactions (F (4.743, 1327.962) = 103.18, *p* <.001, η^2^_p_ =.849; BF = + ∞). TP (*p* <.001; d _std_ = 2.917; BF = + ∞) and FP responses (*p* <.001; d _std_ = 2.845; BF = + ∞) showed significance, TN (*p* =.456; d _std_ =.01; BF =.17) and FN (*p* =.447; d _std_ =.011; BF =.16) did not. Females (M = 4.011, SD =.162) showed significantly more negative scores for valence compared to males (M = 4.987, SD =.167; *p* <.001; d _std_ = 6.051; BF = + ∞). These results showed hit-rate adjustments provided evidence for changes in self-report ratings, but these changes were restricted to instances when participants perceived, or erroneously believed that they perceived, an emotional elicitor (Fig. 2).

## Experiment Two: Temporal Primacy and Psychophysiology

*Objectives*: The objectives of this experiment were to confirm whether overt emotional faces were effective elicitors for psychophysiological responses and participant self-reports, and, subsequently, assess self-reports for time-scale analyses to determine whether physiological experience or conscious awareness took temporal precedence in the processing of the presented visual elicitors.

*Participants*: 282 participants were required for this experimental study when applying the same statistical power calculation parameters as Experiment One. In a preliminary session, 298 volunteers were screened. The preliminary session included the same duration, design, assessments and exclusion criteria as Experiment One. Eleven participants who fitted the criteria for inclusion did not volunteer to further participate in the main study. Data from three participants were excluded from further analysis due to SPHRQ scores that indicated a possible psychiatric diagnosis. Data from a single participant were excluded due to scores in both the SLESQ and MIQ questionnaires that indicated a possible post-traumatic disorder. Data from a single participant were excluded due to AQ scores that indicated Alexithymia traits. The population sample consisted of 282 participants (141 females) with mean age 27.89 (SD = 4.52) years. The mean age for males and females was 28.77 (SD = 4.88) and 27.01 (SD = 4.16) respectively. No participant who took part in any stage or session of Experiment One was included in any part of experiment two. The experiment was approved by the Ethics Committee of the School of Psychology of the University of Nottingham.

**Fig. 3:**
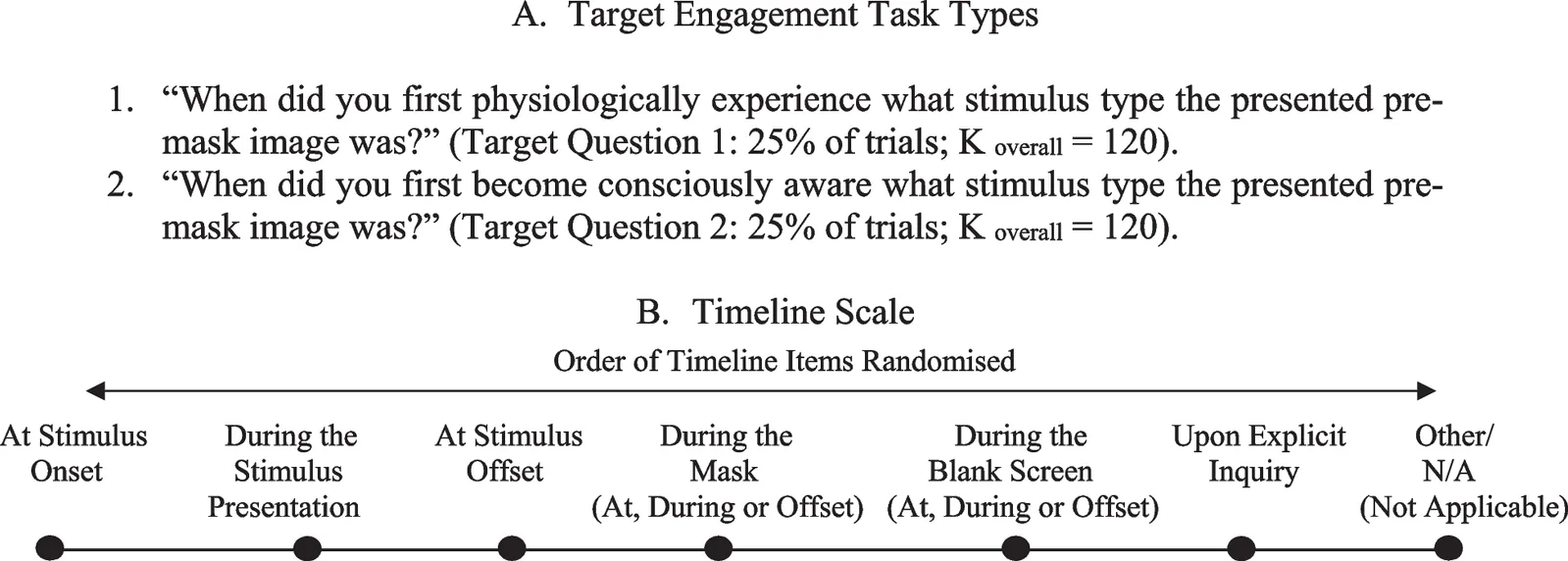
Experiment two, time-scale assessment. The timescale series engagement task presented to the participants during Experiment Two. The participants were briefed and allowed to ask questions concerning any item or aspect of the timescale, and the experimental study. The items and phrasing of the timescale were controlled for and selected using Rasch instrument development analysis in previous publications by the current group. The time-scale items described above will be abbreviated in further mentions, in order of timescale order, as ATO, DSP, ASO, DM, DBS, UEI and Other (see Tsikandilakis et al., 2025c; 2026)

*Experimental Procedures*: In the main experiment, each trial started with an on-screen fixation cross for two (± one) seconds. After the fixation cross, a single face showing fear or anger or happiness or sadness, or a neutral expression, or a blur was presented at fixation for one second; order randomized (k _facial type_ = 48; k _facial type/gender_ = 24; k _blurs_ = 240; K _overall_ = 480). The image was immediately followed by a black-and-white pattern mask for one second. Then a blank screen was presented for five seconds. The psychophysiological assessments and the engagement tasks in Experiment One were included in this experiment (see Tsikandilakis et al., 2019c). The participants were assigned an additional task; order randomised. They were presented with a labelled timeline scale of the experimental trial; timeline series randomised (see Fig. 3A-B). In 25% of the trials (k _facial type_ = 12; k _facial type/gender_ = 6; k _blurs_ = 60; k _overall_ = 120), participants were asked, “When did you first physiologically experience what stimulus type the presented pre-mask image was?”. In 25% of the trials, participants were asked, “When did you first become consciously aware what stimulus type the presented pre-mask image was?”. In 50% of the trials, participants were asked control questions to avoid pre-trial self-invigilation biases (see Proulx et al., 2017; see also Rosati, 2017). In 25% of the control trials, the participants were asked, “When did you last physiologically experience what stimulus type the presented pre-mask image was?”. In 25% of the control trials, participants were asked, “When were you last conscious of what stimulus type the presented pre-mask image was?”. To ensure that participants were attentive to the experiment, after the completion of the experiment, they were presented with two on-screen tasks; order randomised. In one task, they were asked to rate from one (not at all) to nine (very) “How focused were you during the different post-presentation “Experience” and “Conscious Awareness” tasks?”. In one task, all the text-labels of the timescale, and seven variations of the timescale items were presented on-screen. In the variations, the word “stimulus” was made to switch places with the word “image” (e.g., “When were you last conscious of what *image* type the presented pre-mask stimulus was?”), the word “During” was replaced with “Throughout” when the word “stimulus” was not part of the same sentence, and the words “Upon” and “Other” were replaced with the words “On” and “None”. The participants were asked to select which items were included in the experiment (for a detailed review of this methodology, see Wilson & Golonka, 2013; pp. 6–9; Tsikandilakis et al., 2025c; see also Fig. 3A-B).

*Results. Psychophysiology and Self-Reports*: Participants reported perceiving 99.99% (SD ≤.01%; A =.99; SD ≤.01) of the emotional faces, rated their attendance to target-tasks with high confidence (M = 8.67; SD =.073), and selected the correct answers with high accuracy for which tasks were included in the experiment (HRs = 99.99%; SD =.01%; A =.99; SD ≤.01), showing that stimuli were presented overtly, and the target-tasks were appropriately attended. For SCR, significant differences were shown among Stimulus Types (F (4.674, 1308.729) = 4699.694, *p* <.001, η^2^_p_ =.962; BF = + ∞) and a Gender interaction (F (4.674, 1308.729) = 179.195, *p* <.001, η^2^_p_ =.39; BF = + ∞). Females (M =.268, SD =.026) showed higher SCR compared to males (M =.225, SD =.029; *p* <.001; d _std_ = 1.561; BF = + ∞). Similar results were revealed for heart-rate responses for Stimulus Types (F (3.136, 878.027) = 13,329.002, *p* <.001, η^2^_p_ =.979; BF = + ∞) and a Gender interaction (F (3.136, 878.027) = 319.609, *p* <.001, η^2^_p_ =.533; BF = + ∞). Females (M = 9.52, SD =.908) showed higher scores compared to males (M = 7.472, SD =.807; *p* <.001; d _std_ = 2.384; BF = + ∞). Noldus FaceReader showed significant differences for Stimulus Types (F (4.665, 1306.173) = 4494.259, *p* <.001, η^2^_p_ =.941; BF = + ∞) and a Gender interaction (F (4.665, 1306.173) = 32.211, *p* <.001, η^2^_p_ =.103; BF = + ∞). Females (M =.701, SD =.063) showed higher scores compared to males (M =.657, SD =.054; *p* <.001; d _std_ =.801; BF = + ∞). For intensity, significant differences were shown among Stimulus Types (F (4.728, 1294.703) = 7817.127, *p* <.001, η^2^_p_ =.977; BF = + ∞) and a Gender interaction (F (4.728, 1294.703) = 84.325, *p* <.001, η^2^_p_ =.231; BF = + ∞). Females (M = 6.497, SD =.327) showed significantly higher scores for intensity compared to males (M = 5.499, SD =.308; *p* <.001; d _std_ = 3.142; BF = + ∞). For valence, significant differences were shown for Stimulus Types (F (3.749, 1049.641) = 15.396, *p* <.001, η^2^_p_ =.397; BF = + ∞) and a Gender interaction (F (3.749, 1049.641) = 184.274, *p* <.001, η^2^_p_ =.982; BF = + ∞). Females (M = 4.076, SD =.389) showed significantly more negative scores for valence compared to males (M = 4.769, SD =.354; *p* <.001; d _std_ = 1.863; BF = + ∞). These results showed the stimuli were effective emotional elicitors (Table 1A).

**Table 1:** Psychophysiology and timescale analyses outcomes, experiment two

Physiological and self-report outcomes									
Mean (SD)									
	SCR (μS)	HR (bpm)	Noldus (C.)					Intensity	Valence
			Anger	Fear	Happy	Sad	Neutral		
Anger	.372 (.041)*	16.752 (1.968)*	.758 (.014)*	.245 (.021)^H0^	.278 (.02)^H0^	.201 (.021)^H0^	.201 (.021)^H0^	8.234 (.32)*	1.844 (.463)*
Fear	.324 (.032)*	13.509 (2.795)*	.233 (.022)^H0^	.754 (.016)*	.279 (.02)^H0^	.203 (.021)^H0^	.201 (.022)^H0^	7.955 (.274)*	2.105 (.481)*
Happy	.324 (.045)*	13.56 (2.652)*	.223 (.02)^H0^	.245 (.021)^H0^	.752 (.017)*	.214 (.022)^H0^	.201 (.02)^H0^	7.964 (.309)*	8.159 (.396)*
Sad	.185 (.058)*	2.695 (.551)*	.223 (.021)^H0^	.210 (.022)^H0^	.219 (.021)^H0^	.753 (.016)*	.201 (.02)^H0^	3.142 (.508)*	2.77 (.774)*
Neutral	.137 (.03)*	2.21 (.295)*	.222 (.023)^H0^	.209 (.023)^H0^	.287 (.024)^H0^	.201 (.021)^H0^	.753 (.011)*	5.858 (.333)*	5.393 (.339)^*^
Timescale of stimulus-type experience outcomes (%)									
	Angry	Fear	Happy	Sad	Neutral	Overall			
ATO	78.129 (3.682)*	78.515 (3.684)*	78.258 (3.609)*	78.154 (3.693)*	78.154 (3.621)*	78.394 (3.658)*			
DSP	3.46 (.678)^H0^	3.384 (.673)^H0^	3.435 (.643)^H0^	3.459 (.659)^H0^	3.456 (.68)^H0^	3.418 (.667)^H0^			
ASO	3.427 (.452)^H0^	3.456 (.449)^H0^	3.421 (.429)^H0^	3.484 (.44)^H0^	3.484 (.453)^H0^	3.428 (.445)^H0^			
DM	3.423 (.377)^H0^	3.379 (.374)^H0^	3.501 (.358)^H0^	3.459 (.366)^H0^	3.459 (.378)^H0^	3.437 (.37)^H0^			
DBS	3.431 (.314)^H0^	3.399 (.312)^H0^	3.424 (298)^H0^	3.443 (.305)^H0^	3.443 (.315)^H0^	3.412 (.309)^H0^			
UEI	3.445 (.393)^H0^	3.399 (39)^H0^	3.429 (.372)^H0^	3.441 (.382)^H0^	3.441 (.393)^H0^	3.414 (.386)^H0^			
Other	4.687 (.589)**	4.466 (584)**	4.539 (.559)**	4.562 (.572)**	4.56 (.59)**	4.498 (.579)**			
Timescale of stimulus-type awareness outcomes (%)									
	Angry	Fear	Happy	Sad	Neutral	Overall			
ATO	3.45 (.487)^H0^	3.338 (.48)^H0^	3.535 (.477)^H0^	3.41 (.482)^H0^	3.451 (.501)^H0^	3.437 (.486)^H0^			
DSP	78.435 (4.209)*	78.526 (4.032)*	77.63 (4.016)*	78.755 (4.133)*	78.425 (4.443)*	78.354 (4.167)*			
ASO	3.491 (.569)^H0^	3.378 (.56)^H0^	3.509 (.556)^H0^	3.405 (.563)^H0^	3.443 (.584)^H0^	3.445 (.566)^H0^			
DM	3.456 (.474)^H0^	3.349 (.467)^H0^	3.573 (.463)^H0^	3.401 (.469)^H0^	3.461 (.487)^H0^	3.448 (.472)^H0^			
DBS	3.5 (.396)^H0^	3.426 (.392)^H0^	3.617 (.39)^H0^	3.402 (.391)^H0^	3.436 (.406)^H0^	3.476 (.395)^H0^			
UEI	3.502 (.987)^H0^	3.383 (.972)^H0^	3.585 (.965)^H0^	3.401 (.978)^H0^	3.436 (1.105)^H0^	3.461 (.983)^H0^			
Other	4.167 (.494)**	4.6 (.486)**	4.551 (.482)**	4.227 (.489)**	4.331 (.508)**	4.375 (.492)**			

In A., B. and C., the outcomes for physiology, self-reports and timescale analyses. The abbreviations in B. and C. are explained in Fig. 3. * (Asterisk) shows Bonferroni-corrected significance at *p* ≤ .001. ** (Double asterisk) shows significance at .001 < *p* ≤ .01. ^H0^ (superscript) signifies evidence for equivalence of significance to the null

*Results. Timescale Analyses*: For Target Question 1 (Experience), there were significant differences among Time-Scale Items (F (6, 1680) = 273120.164, *p* <.001, η^2^_p_ =.999; BF = + ∞; see Fig. 3) and a significance trend for differences among Stimulus-Types (F (2.21, 1120) = 2.467, *p* =.0791, η^2^_p_ =.009; BF = 1.78). Bonferroni corrections showed that for all stimulus types, timeline item “At Stimulus Onset” was higher than all other items combined (*p* <.001; d _std_ = 28.973; BF = + ∞), and most critically higher than timeline item “During the Stimulus Presentation” (*p* <.001; d _std_ = 28.516; BF = + ∞). For Target Question 2 (Awareness), we showed significant differences among Time-Scale Items (F (6, 1680) = 220793.848, *p* <.001, η^2^_p_ =.999; BF = + ∞) and Stimulus-Types (F (3.961, 1109.043) = 3.621, *p* =.006, η^2^_p_ =.13; BF = + ∞). Bonferroni corrections showed that for all stimulus types, timeline item “During the Stimulus Presentation” was higher than all other items combined (*p* <.0001; d _std_ = 19.071; BF = + ∞), and most critically timeline item “At Stimulus Onset” (*p* <.001; d _std_ = 25.254; BF = + ∞). These findings showed strong evidence that the experience of the type of the presented elicitor preceded conscious awareness (Table 1B-C).

## Experimental Three: Priming and Inhibition

*Objectives*: The objectives of this experiment were to explore whether explicit instructions for priming, and inhibition could induce psychophysiological and behavioural changes to participants’ responses to overt emotional faces.

*Participants*: 322 participants were required for this experimental study when applying the same statistical power calculation parameters as Experiments One and Two. The exclusion criteria were identical to Experiments One and Two. In a preliminary session, 356 volunteers were screened. The preliminary session included was identical to Experiments One and Two. Twenty-nine participants who fitted the criteria for inclusion did not volunteer to further participate in the main study. Data from three participants were excluded from further analysis due to SPHRQ scores that indicated a possible psychiatric diagnosis. Data from two participants were excluded due to AQ scores that indicated Alexithymia traits. The population sample consisted of 322 participants (161 females) with mean age 26.54 (SD = 6.71) years. The mean age for males and females was 26.23 (SD = 6.49) and 26.85 (SD = 6.93) respectively. No participant who took part in any stage or session of Experiments One and Two was included in any part of the experiment three. The experiment was approved by the Ethics Committee of the School of Psychology of the University of Nottingham.

*Experimental Procedures*: In this experiment, each trial started with an on-screen fixation cross for two (± one) seconds. After the fixation cross, a single face showing fear or anger or happiness or sadness, or a neutral expression, or a blur was presented at fixation for one second; order randomized (k _facial type_ = 48; k _facial type/gender_ = 24; k _blurs_ = 240; k _overall_ = 480). The image was immediately followed by a black-and-white pattern mask for one second, and then a blank screen was presented for five seconds.

Additionally, the presentation was divided into four even trials conditions. In one condition (Condition One, Blank: 25% of trials; k _facial type_ = 12; k _facial type/gender_ = 6; k _blurs_ = 60; k _overall_ = 120), a blank screen was presented before each trial. In one condition (Condition Two, Present), a text reading “A stimulus will be presented” was shown on-screen before each trial. In one condition (Condition Three, Focus; Target Condition: Priming), a text reading “A stimulus will be presented, try to focus on the experience of the presentation” was shown before each trial. In one condition (Condition Four, Target condition: Inhibition), a text reading “A stimulus will be presented, try to inhibit the experience of the presentation”. All conditions lasted for three seconds with order of presentation randomised. The engagement tasks and further experimental assessments and intervals sequence included in Experiment One – but not the timescale task included in experiment two – were repeated in this experiment. After the completion of the experiment, participants were presented with two on-screen tasks; order randomised. In one task, they were asked to rate from one (not at all) to nine (very) “How focused were you during the pre-image text presentation?”. In one task, all the text-conditions included in this experiment and four variations of the text-conditions, in which the phrases “ A stimulus” were replaced by “An image”, were presented on-screen. The participants were asked to select which text-conditions were included during the experiment (see Tsikandilakis et al., 2025c; see also Table 2).

**Table 2:** Multivariate outcomes, experiment three

	Mean (SD)								
	SCR	HR	Noldus FaceReader					Intensity	Valence
			Anger	Fear	Happy	Sad	Neutral		
Condition 1: Blank									
Angry	.332 (.036)^H0^	13.837 (1.255)^H0^	.747 (.016)^H0^	.245 (.021)^H0^	.248 (.02)^H0^	.251 (.021)^H0^	.239 (.021)^H0^	7.825 (.48)^H0^	1.954 (.163)^H0^
Fear	.379 (.026)^H0^	16.236 (1.063)^H0^	.249 (.022)^H0^	.748 (.017)^H0^	.247 (.022)^H0^	.241 (.021)^H0^	238 (.023)^H0^	8.12 (.489)^H0^	2.025 (.282)^H0^
Happy	.332 (.035)^H0^	13.841 (1.333)^H0^	.251 (.019)^H0^	.25 (.021)^H0^	.749 (.014)^H0^	.248 (.02)^H0^	.239 (.021)^H0^	7.826 (.508)^H0^	7.616 (.693)^H0^
Sad	.157 (.017)^H0^	3.155 (.317)^H0^	.248 (.021)^H0^	.245 (.022)^H0^	.231 (.021)^H0^	.749 (.015)^H0^	.238 (.021)^H0^	4.086 (.523)^H0^	2.585 (.497)^H0^
Neutral	.112 (.017)^H0^	2.23 (.293)^H0^	.245 (.021)^H0^	.247 (.02)^H0^	.249 (.022)^H0^	.245 (.021)^H0^	.784 (.013)^H0^	5.13 (.574)^H0^	5.131 (.595)^H0^
Condition 2: Present									
Angry	.332 (.035)^H0^	13.841 (1.406)^H0^	.748 (.015)^H0^	.245 (.021)^H0^	.246 (.023)^H0^	.243 (.021)^H0^	.245 (.021)^H0^	7.826 (.475)^H0^	1.957 (.167)^H0^
Fear	.38 (.026)^H0^	16.233 (1.102)^H0^	.245 (.021)^H0^	.747 (.016)^H0^	.241 (.021)^H0^	.245 (.02)^H0^	.246 (.022)^H0^	8.12 (.516)^H0^	2.031 (.303)^H0^
Happy	.332 (.037)^H0^	13.838 (1.338)^H0^	.244 (.02)^H0^	.244 (.021)^H0^	.746 (.018)^H0^	.243 (.021)^H0^	.245 (.022)^H0^	7.827 (.494)^H0^	7.617 (1.072)^H0^
Sad	.158 (.017)^H0^	3.129 (.314)^H0^	.246 (.02)^H0^	.245 (.021)^H0^	.245 (.022)^H0^	.748 (.016)^H0^	.244 (.021)^H0^	4.087 (.462)^H0^	2.582 (.479)^H0^
Neutral	.113 (.017)^H0^	2.264 (.295)^H0^	.247 (.021)^H0^	.248 (.022)^H0^	.247 (.02)^H0^	.241 (.021)^H0^	.782 (.018)^H0^	5.117 (.58)^H0^	5.087 (.614)^H0^
Condition 3: Focus									
Angry	.378 (.028)*	15.943 (1.232)*	.785 (.015)*	.248 (.022)^H0^	.249 (.021)^H0^	.248 (.02)^H0^	.249 (.023)^H0^	8.089 (.867)*	1.594 (.241)*
Fear	.412 (.028)*	18.054 (1.209)*	.248 (.021)^H0^	.785 (.014)*	.251 (.019)^H0^	.247 (.02)^H0^	.245 (.021)^H0^	8.18 (.559)*	1.675 (.499)*
Happy	.376 (.021)*	15.933 (1.234)*	.247 (.021)^H0^	.245 (.022)^H0^	.784 (.016)*	.248 (.021)^H0^	.248 (.02)^H0^	8.076 (.875)*	8.01 (.593)*
Sad	.207 (.018)*	4.029 (.338)*	.245 (.021)^H0^	.247 (.02)^H0^	.25 (.022)^H0^	.783 (.015)*	.246 (.021)^H0^	3.656 (.633)*	2.234 (.295)*
Neutral	.113 (.016)^H0^	2.262 (.298)^H0^	.247 (.021)^H0^	.248 (.022)^H0^	.249 (.02)^H0^	.246 (.021)^H0^	.783 (.013)*	5.13 (.586)^H0^	5.077 (.616)^H0^
Condition 4: Inhibition									
Angry	.332 (.058)^H0^	13.861 (1.593)^H0^	.238 (.021)*	.241 (.023)^H0^	.239 (.021)^H0^	.239 (.021)^H0^	.782 (.013)*	6.828 (.717)*	2.953 (.17)*
Fear	.384 (.028)^H0^	16.227 (1.285)^H0^	.241 (.021)^H0^	.236 (.022)*	.238 (.021)^H0^	.239 (.02)^H0^	.779 (.012)*	7.119 (.632)*	3.023 (.332)*
Happy	.332 (.059)^H0^	13.845 (1.566)^H0^	.241 (.019)^H0^	.242 (.021)^H0^	.239 (.02)*	.241 (.022)^H0^	.781 (.012)*	6.827 (.718)*	6.602 (.615)*
Sad	.158 (.016)^H0^	3.163 (.333)^H0^	.243 (.021)^H0^	.241 (.02)^H0^	.24 (.021)^H0^	.243 (.022)*	.776 (.021)*	5.083 (.809)*	3.567 (.488)*
Neutral	.112 (.017)^H0^	2.241 (.284)^H0^	.241 (.022)^H0^	.239 (.021)^H0^	.242 (.02)^H0^	.241 (.021)^H0^	.783 (.011)^H0^	5.158 (.551)^H0^	5.124 (.594)^H0^

Overall summary of outcomes for experiment three. Asterisks (*) show Bonferroni-corrected significant differences (*p* ≤.001) for an item compared among conditions to their respective stimulus type. ^HO^ (superscript) show items that provided Bayesian evidence for equivalence of significance to the null (BF <.33) that they were proximate among conditions to their respective stimulus type

*Results. Psychophysiology and Self-Reports*: Participants reported perceiving 99.99% (SD ≤.01%; A =.99; SD ≤.01) of the emotional faces, rated their attendance to target-tasks with high confidence (M = 8.79; SD =.064), and reported high metrics for choosing the correct answers for which tasks were included in this experiment (HRs = 99.99%; SD =.01%; A =.99; SD ≤.01). For SCR, there were overall significant differences among Conditions (F (1.883, 602.657) = 651.708, *p* <.001, η^2^_p_ =.671; BF = + ∞) and Stimulus Types (F (2.611, 835.55) = 39218.907, *p* <.001, η^2^_p_ =.992; BF = + ∞) and a significant interaction among Conditions and Stimulus Types (F (4.992, 1597.509) = 83.9, *p* <.001, η^2^_p_ =.208; BF = + ∞). The same pattern applied for HR for differences among Conditions (F (2.258, 722.614) = 638.558, *p* <.001, η^2^_p_ =.666; BF = + ∞) and Stimulus Types (F (1.843, 589.722) = 59527.74, *p* <.001, η^2^_p_ =.995; BF = + ∞) including a significant interaction among Conditions and Stimulus Types (F (5.551, 1776.173) = 137.733, *p* <.001, η^2^_p_ =.301; BF = + ∞). The Noldus assessment showed significant differences among Conditions (F (2.727, 872.545) = 12330,617 *p* <.001, η^2^_p_ =.975; BF = + ∞) and Stimulus Types (F (3.956, 1265.951) = 1938.308, *p* <.001, η^2^_p_ =.86; BF = + ∞) and a significant interaction among Conditions and Stimulus Types (F (11.434, 3658.844) = 1117.516, *p* <.001, η^2^_p_ =.761; BF = + ∞).

Ratings for intensity showed significant differences among Conditions (F (2.507, 804.639) = 38.058, *p* <.001, η^2^_p_ =.131; BF = + ∞) and Stimulus Types (F (1.704, 547.002) = 11520.683, *p* <.001, η^2^_p_ =.973; BF = + ∞) and a significant interaction among Conditions and Stimulus Types (F (5.962, 928.664) = 26.358, *p* <.001, η^2^_p_ =.076; BF = + ∞). The same applied for valence that showed significant differences among Conditions (F (2.792, 896.348) = 40.106, *p* <.001, η^2^_p_ =.135; BF = + ∞) and Stimulus Types (F (1.67, 535.943) = 19237.008, *p* <.001, η^2^_p_ =.984; BF = + ∞) and a significant interaction among Conditions and Stimulus Types (F (5.708, 1832.142) = 50.421, *p* <.001, η^2^_p_ =.136; BF = + ∞).

These differences were reported among Conditions 1–2, and 4, and Conditions 3–4 for Stimulus Types. Conditions 1–2 and 4 provided Bayesian evidence (BF <.33) for proximal responses for SCR and HR. Condition 3 was significantly higher for all responses, and Condition 4 significantly lower than any other condition only for explicit responses, i.e., facial-expressions and self-report attractiveness ratings. These results showed that self-reports and facial expressions could be primed and inhibited, while peripheral nervous system responses could be primed, but not inhibited, by conscious instructions prior to the presentation of emotional faces (see Table 2).

## Discussion

### Summary and Synopses

In this set of studies, we explored the current paradigm for the unconscious in experimental psychology. Specifically, we explored two models of unconscious PNS and behavioural responsivity: *Subliminal processing, and perception*, and the applied elements of the conceptual paradigm of *The Unconscious Mind*. We employed an individual-thresholds model for exploring responses to imperceptible elicitors for subliminality, and timescale analyses, and priming and inhibition conditions for testing the validity of the tenets and requirements for an interactive unconscious with temporal primacy to conscious awareness. In Experiment One, we used Bayesian inference and successfully adjusted masked presentations to chance-level detection performance individually for each participant and stimulus type. When these adjustments were made using unbiased sensitivity index A, we did not report physiological, or behavioural differences among different types of masked emotional faces. When these adjustments were made using hit rates, we reported significant differences among emotional faces. Further analyses showed that these differences were restricted to TP and FP responses, and that FN responses did not provide NHST outcomes for different responses, and provided Bayesian outcomes for null responses among stimulus types. In Experiments Two and Three, we showed that perceptible emotional faces were effective physiological and behavioural elicitors. In Experiment Two, we showed using timescale analyses that participants reported that physiological experience preceded the conscious perception of emotional faces. In Experiment Three, we showed that explicit instruction for emotional inhibition did not restrict PNS arousal, but was sufficient to induce behavioural inhibition, such as showing null responses for facial-emotional expressions. Interestingly, we showed an opposite pattern of results for explicit instructions for attentional focus. This priming condition showed significantly enhanced stimulus-type related changes in physiological and facial-emotional expression responses, and self-reports. The results showed that we could not support the notion of subliminal processing. They showed that the conceptual paradigm of the unconscious mind could apply to explain our spontaneous and initially uninhibited unconscious, and automatic and involuntary PNS and behavioural responses, and their further interactions with conscious perception and awareness.

### It is Not Subliminal – The Provocation

In Experiment One, we were able to successfully adjust individual thresholds for subliminal presentations for each participant for each stimulus type. We provided Bayesian evidence for equivalence to the null that participants performed at chance level for signal detection (Dienes, 2021a), using sensitivity index A and hit rates (Hautus et al., 2021). As per our hypotheses, we showed that for α = 0, using unbiased sensitivity index A, we did not observe NHST differences and observed Bayesian evidence for null differences among different emotional faces. When using hit rates, we showed that there were differences in emotional responses among different emotional faces. These differences were significant only for TP, such as correctly responding that a face was presented, and FP responses, such as incorrectly responding that a face was presented. Additionally, using hit rates, FN responses, such as incorrectly responding that a face was not presented, did not provide NHST evidence, and provided Bayesian evidence for equivalence to the null, for physiological and behavioural changes (see Pessoa et al., 2006; pp. 369–371; see also Pessoa, 2005, 2010, 2017). Importantly, this pattern of results was reported without exception for SCR, heart-rate responses, facial-emotional expression responses, and valence and intensity self-reports (see Tsikandilakis et al., 2020a).

These findings showed that using objective measures for null perception (Macmillan, 2002), the psychophysical framework of subliminal perception, such as ε > α | α = 0, did not result in physiological and behavioural outcomes (see Overgaard & Timmermans, 2009; pp. 501–503). Conversely, when using potentially biased measures for null perception (Stanislaw & Todorov, 1999), exhaustive and exclusive analyses showed that emotional changes were restricted to trials in which participants reported either conscious perception (TP), or the impression of conscious perception (FP), of an emotional face (see Overgaard & Timmermans, 2009; pp. 504–7). Therefore, we were not able to provide supporting evidence for subliminal processing, such as emotional changes to imperceptible emotional faces. Instead, we thoroughly, and exhaustively and exclusively provided evidence that emotional changes involved either conscious perception, or the impression of conscious perception of an emotional elicitor (see Tsikandilakis et al., 2025b). These findings strongly suggested that, as a model, subliminal processing, and by extension subliminal perception (see Pessoa & Adolphs, 2010; pp. 3–7), could not apply to explain whether and how unconscious PNS and behavioural responses operate. We claim, as provocatively as it should be stated, that we have thoroughly, and after meeting every requirement included in the contemporary state-of-the-art methodological and analytical experimental/empirical-testing of the unconscious, disproved that imperceptible stimuli can be effective PNS and behavioural elicitors, and that we have disproved the hard model of subliminality, and subliminal processing as presented in contemporary psychophysics (see Hesse et al., 2016; pp. 504–7; Sand & Nilsson, 2016; pp. 31–3; but see also Elgendi et al., 2018; pp. 7–11).

#### It can be Unconscious – “The Remaking…”

In Experiment Two, we validated with sensitivity index A and hit rates, and catch trials that the stimuli were visible and attended. We validated that the stimuli were efficient emotional elicitors using SCR, heart-rate responses, facial-emotional expression analyses, and self-reports. We accomplished two fundamentals, for further experimentation, tenets of the unconscious mind model: α > 0 and ε > 0. We employed a timescale task to explore another fundamental tenet of the unconscious mind model: We explored whether the physiological experience of emotion preceded conscious perception and awareness, such as ε_τ(x)_ ≺ α_τ(y)_ | α > 0. The participants reported strong evidence that the physiological experience of emotion preceded conscious perception and awareness. Their responses were almost exclusively divided, both for physiological experience, and conscious perception and awareness, between the first two items of the timescale; i.e., “at-stimulus-onset” and “during-stimulus-presentation”. This showed that, although physiological experience was faster than conscious perception and awareness, conscious awareness was not a slow post-trial mechanism. It was an arguably faster than previously reported (i.e., ≥ one second; for relevant reviews, see Engel & Singer, 2001; Engelen & Mennella, 2023; Libet, 2005; Rabbitt, 2019), mechanism with possibly dedicated sociobiological, and dual-processing, interactive cognitive-appraisal and reappraisal, actionable and skill-acquisition value, such as social-adaptation and memory-recall practical-applied significance respectively. As such, we claim, as provocatively as it can possibly be stated, that we have provided evidence for a refined paradigm of “The Unconscious Mind” that satisfies the contemporary psychophysics of the temporal primacy of unconscious experience over conscious awareness: When exposed to emotional content, we feel first, we consider afterwards – even if the temporal difference of these interactive functions are very brief, and almost exclusively clustered in the very first milliseconds, or seconds of our experience (see Dehaene & Changeux, 2011; pp. 201–9; see also Evans & Stanovich, 2013).

Along these lines, it is also worth discussing that the results of Experiment Two point towards a previously unaddressed and seminal dissociation between the unconscious mind model and preconscious process-mechanisms (see Dehaene et al., 2006; pp. 206–9). Preconscious processes are suggested to occur when an individual has latent conscious access, and available experiential accessibility to an emotional elicitor. In Experiment Two, we showed strong evidence for differences to these process-mechanisms: Unconscious emotion occurred before, but not without eventual active and manifest conscious access to, and meta-awareness and meta-cognition of an emotional elicitor. We presented evidence that experiential accessibility as shown to operate via the unconscious mind processing mechanisms happened before, and, therefore, without initial access and accessibility to conscious awareness. The workings of preconscious processing are suggested to involve experiential accessibility, and simultaneous latent and available access to conscious awareness. Our results did not show preconscious responses – in the sense that the initial experience of emotion was latently accessible by conscious awareness – they showed unconscious responses – in the sense that unconscious experience preceded access and accessibility to conscious awareness (see Tzelgov, 2014; pp. 221–4; Boag, 2010; pp.170–1).

In Experiment Three, we reported that responses to emotional faces could be primed to change by simply asking participants to focus on the presentation. Nevertheless, the participants could not inhibit, even upon explicit instruction, their automatic and involuntary PNS responses, such as SCR and heart-rate responses. Additionally, the participants were able to inhibit their facial-emotional expression responses. These results are in line with the dual-processing pathways model that is suggested to comprise the neural components of an intelligent and interactive theory of the unconscious mind. The dorsal pathway of the unconscious mind model is suggested to exert prefrontal cortex induced control over behavioural responses, such as facial-emotional expressions. These responses could be restricted upon explicit instruction for inhibition. On the other hand, the ventral pathways of the unconscious mind model are suggested to involve PNS correlates that occur through a neural cascade from the occipital cortex to the temporal lobe and then the brainstem and the cerebellum. These emotional processing loci operate the dissemination of automatic and involuntary arousal in response to emotional elicitors. The downstream output of these structures cannot be regulated by executive control functions. These responses could not be restricted upon explicit instruction for inhibition. These characteristics point towards a confirmation of a set of fundamental tenets of the concept of unconscious mind model: Automatic and involuntary PNS responses could not be inhibited by conscious awareness, possibly because they have important sociobiological and survival-related value; behavioural responses, such as facial-emotional expressions, could be inhibited by conscious instruction, and automatic and involuntary PNS responses could be primed to increase by conscious instruction, possibly because the latter two can also function to accommodate survival-related and sociobiological purposes, such as increased emotional arousal and behavioural inhibition when these are required by real-life demands in specific ecological contexts (see Tsikandilakis et al., 2025a, 2026). These provided support that the unconscious mind model is valid, and could be applied to explain our unconscious, and automatic and involuntary PNS, and behavioural responses, and their interactions with conscious awareness (see Boag, 2020; pp. 859–861).

#### It can be Unconscious – “… of A Paradigm”

In the same spirit that this manuscript begun, here we claim again that the evolutionary dynamics of subliminality are explicitly reflected in the current empirical results: Subliminal processing does not and cannot occur; it is paradoxical. Firstly, as stated emphatically before (Overgaard & Timmermans, 2009), real life does not involve masked-imperceptible emotional elicitors. We have never, we will never, and even if we could, we would not be able to know that we encountered a real-life imperceptible elicitor (see Boag, 2020). This brings us to an evolutionary paradox: Subliminal processing requires that a significant and physiological effective imperceptible emotional elicitor will result in significant imperceptible physiological changes (Bargh & Morsella, 2008). The second part of this requirement is unreasonable, in the sense that self-perception will result in some form of awareness of changes in our physiological homeostasis during physiological experience, and has been noted as being unreasonable from Heraclitus to Charles Darwin and to Aaron Beck (see Tsikandilakis et al., 2026). From a strictly evolutionary perspective, if at some archaic era we experienced emotion exclusively without consciousness, we have evolved from this hurdle, or consciousness would have never emerged, and we would have not been able to adapt as a species (see Tsikandilakis et al., 2025a, 2025b, 2025c), and refine our behaviour and strategy-guidance systems (see Tsikandilakis et al., 2026).

As provocatively as possible, if imperceptible experience without consciousness was a module, or the whole of our emotional apparatus, we would not be able to manifest consciousness (Baars et al., 2003). If some kind of unconscious response without awareness – that should no longer be referred to as subliminal – could exist it would require to put experiential awareness, and meta-awareness, in a transient Heraclitan *slumber* by the introduction of a distracting task, a fast-sequence of environmental demands, physical exhaustion and cognitive-reserve depletion, or by deference to another elicitor (see Tsikandilakis et al., 2025a, 2026), and even in these cases experiential imperceptibility would last for the duration of these distractions (Bargh & Chartrand, 2000); this is not impossible, but it is not subliminal (Erdelyi, 2004).

On the other hand, we claim that concepts related to the unconscious mind framework make simple reasonable sense, and confer scientific and evolutionary-theory validity. The current results support the simple reality, encountered very frequently in real life: When we are exposed to a significant emotional elicitor or event, it is explicit – or that is what we can possibly know about it (see Erdelyi, 2004; pp. 81–83) – and it elicits emotion, and conscious awareness of emotion – in that order (see Tsikandilakis et al., 2025c, 2026). That should not be interpreted to signify that cognitions, ideas, ideations, and generally, as colloquially as possible, “thinking” cannot induce emotion in the absence of direct exposure to an elicitor. It signifies that when an emotional elicitor or circumstance is presented, it is biologically reasonable to experience first, and think later, with the exception of habituation, conditioning, and repeated exposure, that would not lead to emotion anyway, or lead to already cognitively-framed emotional responses (Donald, 2001). From a strictly evolutionary perspective, the primacy of experience over awareness, serves as a response-mechanism to potential demands for spontaneous action. The eventual recruitment of awareness and cognition serves as a refinement mechanism of these responses. The inability to inhibit experience, and the ability to inhibit expression serve as a necessary drive for responsivity, and a stop-mechanism for non-responsivity with sociobiological value, such as not displaying negative emotions in restricted-emotional-response-range social circumstances, respectively (see Tzelgov, 2014).

As provocatively as possible, we have provided empirical evidence for a series of conceptual tenets: When we encounter emotion in real life, it is explicit and perceivable; we experience emotion first and we have conscious awareness of the emotional elicitor, and our emotional responses to an emotional elicitor; these emotional responses cannot, unless habituation, conditioning, and cognitive-framing are already at work, be inhibited if someone asks of us, “Do not emotional respond to this emotional situation” (see Tsikandilakis et al., 2022, 2023a, 2023b, 2024, 2025a, 2025c, 2026); these are unconscious emotional responses; they will eventually involve conscious awareness (Bargh & Morsella, 2008).

### Concepts and Considerations for Further Research

There are more perspectives on the unconscious than psychological science recognises as strictly canonical at this point in time, and these merit and will be addressed in the subsequent “ACTS” of this series of reforming endeavours (see for example, Kaplan-Solms & Solms, 2018; Shevrin, 1996; Shevrin & Dickman, 1980; Klein Villa et al., 2006). Concerning the current set of paradigms, in the current set of studies, the results could be expanded by replications using additional visual suppression techniques, such as continuous flash suppression and binocular rivalry. They could also benefit from a replication using trial-contour power calculations for validating the current timescale, and inhibition and priming effects using specifically TP and FN responses under conditions of visual suppression although, here, it should be stated very emphatically that this is not part of and it is rejected by the conceptual framework of the unconscious mind paradigm as an ecologically invalid condition (see Bargh & Morsella, 2008; pp. 2–4). An additional consideration is whether the conscious awareness of emotional responses can be influenced by different signal-strength thresholds, and whether it could be modified by repeated exposure, and sufficient partial, graded, or gradual signal strength, such as that not fully imperceptible but partly perceptible elicitors could, for example, at some first threshold of basic awareness implicitly convey more information than immediately and easily accessible by conscious awareness. This is fertile and terra nova area for further empirical explorations (see Fisk & Haase, 2025; Fisk, 2026; Haase, 2026; see also Ramsøy & Overgaard, 2004; Kouider & Dupoux, 2004). Conclusively, it should be highlighted that he timescale implementation and analyses, applied as part of this set of studies, could benefit from avoiding the extensively recorded limitations of Likert-scale self-reports (see Bond et al., 2021; Haralabopoulos et al., 2020; Mével et al., 2025; Leong et al., 2024; Leveridge et al., 2024; Yu et al., 2022), and implemented using state-of-the-art CNS psychophysiological assessments, such as fNIRS and EEG, and fMRI (see Huang, 2021; Murugappan et al., 2010; Russell, 2003; Sohaib et al., 2013; Valenzi et al., 2014; Zheng et al., 2021).

## Conclusions

The current set of studies showed the notion of subliminal processing, and perception, could not be validated. PNS and behavioural responses under conditions of visual suppression occurred only in the subset of trials that involved conscious perception and awareness. In contrast, the tenets of the unconscious mind model could be validated: Timescale analyses showed that experience preceded conscious perception and awareness, and inhibition conditions showed that behavioural, but not PNS responses, could be restricted by conscious instruction. Priming conditions showed that both PNS and behavioural responses could be enhanced by conscious instructions for attentional focus. These results showed that an extended and refined paradigm involving conceptual principles of “The Unconscious Mind” model could be applied to explain our unconscious, and automatic and involuntary PNS and behavioural responses, and their interactions with conscious awareness. Subliminality did not stand the empirical or conceptual tests in was put through; certain concepts of the unconscious mind paradigm did, and pointed towards a novel model-method for the unconscious, therefore, this is merely “Act I” of this laborious endeavour.

## Data Availability

Https://osf.io/75c8u/
